# Laparoscopic and robotic lateral lymph node dissection for rectal cancer

**DOI:** 10.1007/s00595-020-01958-z

**Published:** 2020-01-27

**Authors:** Ryota Nakanishi, Tomohiro Yamaguchi, Takashi Akiyoshi, Toshiya Nagasaki, Satoshi Nagayama, Toshiki Mukai, Masashi Ueno, Yosuke Fukunaga, Tsuyoshi Konishi

**Affiliations:** Department of Gastroenterological Surgery, Cancer Institute Hospital of the Japanese Foundation for Cancer Research, 31-8-3, Ariake, Koto-ku, Tokyo, 135-8550 Japan

**Keywords:** Lateral lymph node dissection, Laparoscopic, Robotic, Rectal cancer

## Abstract

In the era of neoadjuvant chemoradiotherapy/radiotherapy and total mesorectal excision, overall oncological outcomes after curative resection of rectal cancer are excellent, with local recurrence rates as low as 5–10%. However, lateral nodal disease is a major cause of local recurrence after neoadjuvant chemoradiotherapy/radiotherapy and total mesorectal excision. Patients with lateral nodal disease have a local recurrence rate of up to 30%. The oncological benefits of lateral pelvic lymph node dissection (LPLND) in reducing local recurrence, particularly in the lateral compartment, have been demonstrated. Although LPLND is not standard in Western countries, technical improvements in minimally invasive surgery have resulted in rapid technical standardization of this complicated procedure. The feasibility and short- and long-term outcomes of laparoscopic and robotic LPLND have been reported widely. A minimally invasive approach has the advantages of less bleeding and providing a better surgical view of the deep pelvic anatomy than an open approach. With precise autonomic nerve preservation, postoperative genitourinary dysfunction has been reported to be minimal. We review recent evidence on the management of lateral nodal disease in rectal cancer and technical improvements of LPLND, focusing on laparoscopic and robotic LPLND.

## Introduction

Local recurrence of rectal cancer occurs as frequently as liver or lung metastases [1]. Its treatment can be challenging [2] and it impairs quality of life (QOL) with severe pelvic pain, foul-smelling discharge, and neurological disturbance, including tenesmus and incontinence. The Japanese Clinical Oncology Group reported on an RCT that evaluated mesenteric excision (ME) alone vs. ME plus prophylactic lateral pelvic lymph node dissection (LPLND) in patients with cStage II–III low rectal cancer without evident enlargement of the lateral nodes [3]. The 5-year relapse-free survival, being the primary endpoint of the study, was similar in the ME with LLND group and the ME alone group (73.4% and 73.3%, respectively), although the study failed to demonstrate non-inferiority of ME alone. Importantly, the study found a higher local recurrence rate of 12.6% after ME alone vs. 7.4% after ME with LPLND (*p* = 0.024). These data clearly showed the oncological benefit of LPLND for reducing local recurrence of cStage II–III low rectal cancer even without suspicious lateral nodes. That study also identified longer operation times, greater blood loss, and a marginally higher rate of grade 3/4 complications in the ME with LLND group than in the ME alone group (21.7% vs. 16.0%, respectively; *p* = 0.07) [4]. Sexual and urinary dysfunctions were not different in the two groups [5, 6].

**Fig. 1:**
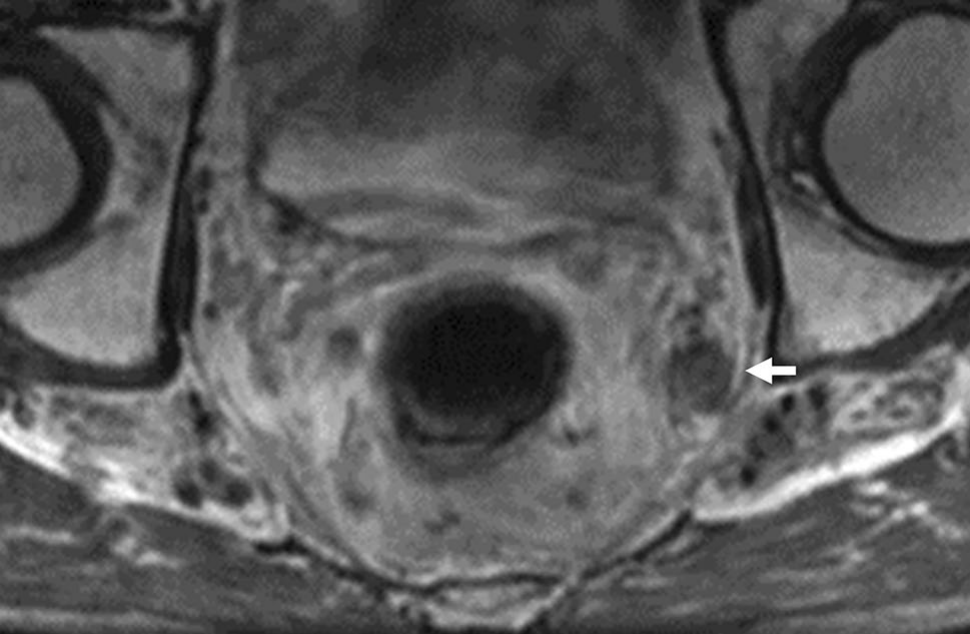
Left lateral node metastasis in the obturator area

In Western countries, neoadjuvant chemoradiotherapy/radiotherapy (NACRT/RT) followed by total mesorectal excision (TME) is standard treatment for cStage II–III rectal cancer [7–9]. Although the overall local recurrence rate with this strategy is 5% to 10% [10, 11], it has been demonstrated that patients with lateral nodal enlargement have a higher rate of local recurrence of up to 30% without LPLND [11]. With improved local control in the central pelvis by NACRT/RT and TME, there is emerging global attention on how to deal with lateral nodal disease as a major cause of local recurrence (Fig. 1) [11, 12]. With recent technical improvements in minimally invasive surgery, studies have demonstrated the safety and feasibility of LPLND through a minimally invasive approach [13, 14]. In this article, we review recent evidence on the management of lateral nodal disease in rectal cancer and technical improvements in LPLND, with particular focus on laparoscopic and robotic LPLND.

Indications for selective lateral node dissection after neoadjuvant therapy. Lateral nodal disease is a major cause of local recurrence after NACRT/RT. In a study from Korea, the local recurrence rate after NACRT and TME reached 26.6% of patients with lateral nodes ≥ 5 mm in diameter and 68.8% of patients with lateral nodes ≥ 10 mm in diameter [15]. A multicenter international study found that lateral local recurrence rates after NACRT/RT in patients with enlarged lateral nodes (≥ 7 mm) were significantly lower in patients who underwent TME plus LPLND than in those who underwent TME alone (5.7% vs. 19.5%, respectively; *P* = 0.042) [11]. These data suggest that preoperative CRT/RT is not sufficient to eliminate lateral nodal disease and that selective LPLND combined with preoperative CRT/RT should be considered for patients with lateral nodal disease.

Although the initial size of the lateral nodes before neoadjuvant therapy remains the gold standard for predicting lateral nodal disease [16], the role of post-treatment nodal size remains controversial. Akiyoshi et al. reported that a short-axis diameter of ≥ 8 mm in the lateral nodes before NACRT, female sex, and NACRT without induction chemotherapy was independently predictive of residual disease in the lateral nodes at final pathology, but that post-treatment size was not predictive [17]. In contrast, Ogura et al. reported the prognostic importance of the post-treatment size of the lateral nodes on restaging magnetic resonance imaging (MRI) after NACRT. Patients with post-treatment lateral nodes < 4 mm had no lateral local recurrence, whereas those with nodes ≥ 7 mm and/or internal iliac nodes ≥ 5 mm had a 5-year lateral local recurrence rate of 52.3% [12]. The authors suggested that LPLND could be avoided for patients with lateral nodes that shrink with treatment.

In addition to node size, Brown et al. showed that the signal intensity and border characteristics of the nodes on MRI were associated with mesorectal nodal involvement [18]. The Mercury Study Group reported that patients with features suspicious of lateral node metastasis on pretreatment MRI, such as a spiculated border and mixed signal intensity, had worse 5-year disease-free survival than other patients (31% vs. 76%, respectively; *P* = 0·001) [19]. These findings suggest that malignant characteristics on MRI could add diagnostic value to the prediction of metastasis. Sex, T stage, histopathological grade, regional lymph node status, PET-CT status, and preoperative induction systemic chemotherapy are potential additional predictive factors to consider instead of the model based on MRI findings alone [17, 20, 21].

### Laparoscopic LPLND

Multiple recent studies have been published on the feasibility of laparoscopic LPLND, short-term outcomes of which have been reported, mainly from Asian countries, since 2011 (Table 1) [13, 22–24]. Although these studies were retrospective case series, the short-term outcomes were reasonable, with median estimated blood [25] loss of 25–213 mL and rates of conversion of 0–17%. Yamaguchi et al. reported a multicenter case-matched study that compared laparoscopic and open LPLND for stage II or III low rectal cancer [26]. They found that the laparoscopic group had a longer operation time (474 min vs. 363 min), less blood loss (213 mL vs. 775 mL), less blood transfusion (6.8% vs. 22.2%), similar rates of grade III or IV complications (23.7% vs. 22.9%), and no mortality. Data on the long-term oncologic outcomes of this procedure are relatively limited. A case series of 107 patients who underwent laparoscopic LPLND after NACRT at a single cancer center in Japan reported 95.8% 3-year overall survival, 84.7% 3-year relapse-free survival, and a 3.2% 3-year local recurrence rate [27]. The patients in that series all had cT3/4 extraperitoneal low rectal cancer with clinically positive lateral nodes; therefore, these data support the oncologic rationale for performing this procedure. A retrospective multicenter case-matched study from Japan, comparing laparoscopic and open LPLND reported 93.9% 3-year overall survival, 93.9% 3-year local recurrence-free survival, and 80.3% 3-year relapse-free survival in the laparoscopic group. These values were all similar to or better than those for the open group [26]. Although prospective validation studies are warranted, these outcomes indicate the technical safety and feasibility of laparoscopic LPLND.

**Table 1:** Laparoscopic lateral pelvic lymph node dissection for rectal cancer

Author	Year	Number of patients	Neoadjuvant chemoradiotherapy %	Operation time (total, min)	Blood loss (total, min)	Number of harvested nodes	Conversion rate %	Overall morbidity %
Liu [22]	2011	68	N/A	271	150	23	N/A	7
Park [23]	2011	16	56	310	188	9	0	31
Liang [24]	2011	34	100	58	44	6	N/A	21
Konishi [13]	2011	14	100	413	25	23	0	36
Bae [25]	2014	21	86	396	200	7	0	29
Ogura [27]	2016	107	100	461	115	25	0	34
Yamaguchi [26]	2017	118	24	474	213	10	17	41
Aisu [28]	2018	25	76	558	100	N/A	0	20.0

*N/A* Not assessed

### Robotic LPLND

Robotic LPLND was first described by Park et al., who reported a series of eight patients [29], since when other authors have documented their results (Table 2) [30–35]. Robotic surgery has the advantages of using multi-joint forceps with a motion scaling, high-quality three-dimensional camera and greatly improved ergonomics, which are all ideal for complex procedures such as LPLND. Yamaguchi et al. reported the short- and long-term oncological outcomes of robotic vs. open LPLND [32, 34]. Robotic LPLND was associated with less blood loss (25 mL vs. 637 mL, *P* < 0.001), less need for blood transfusion (0% vs. 10.2%, *P* = 0.003), longer operative time (455 min vs. 410 min, *P* = 0.007), and fewer postoperative complications (wound infection 0% vs. 8.0%, *P* = 0.014; small bowel obstruction 3.5% vs. 15.9%, *P* = 0.009; anastomotic leakage 0% vs. 9.1%, *P* = 0.007; urinary retention 18.8% vs. 36.4%, *P* = 0.011) than the open procedure. That study also reported similar 5-year overall survival rates (95.4% vs. 87.8%, respectively; *P* = 0.106) and 5-year relapse-free survival rates (79.1% vs. 69.9%, *P* = 0.157) for robotic LPLND and the open procedure, but noted that robotic LPLND had superior 5-year local relapse-free survival (98.6% vs. 90.9%, *P* = 0.029). Kim et al. compared the short-term outcomes of robotic vs. laparoscopic LPLND [35]. Whereas the operative time was similar in the two groups, the estimated blood loss and the incidence of Foley catheter reinsertion for urinary retention after surgery were lower in the robotic group. Overall and local recurrence rates did not differ between the groups. Although there is limited evidence directly comparing robotic and laparoscopic LPLND, a robotic approach is generally regarded as a reasonable alternative for this complicated procedure, particularly in Western countries where LPLND is not standard.

**Table 2:** Robotic lateral pelvic lymph node dissection for rectal cancer

Author	Year	Number of patients	Neoadjuvant chemoradiotherapy %	Operation time (total, min)	Blood loss (total, min)	Number of harvested nodes	Conversion rate %	Overall morbidity %
Park [29]	2012	8	100	272	45	4.1	0	25
Yamaguchi [32]	2016	85	12	455	25	19	0	31
Shin [33]	2016	16	100	401	125	2.5	0	39
Kim [35]	2018	50	86	260	34.6	6.6	0	28

*N/A* Not assessed

### Technical procedures

**Fig. 2:**
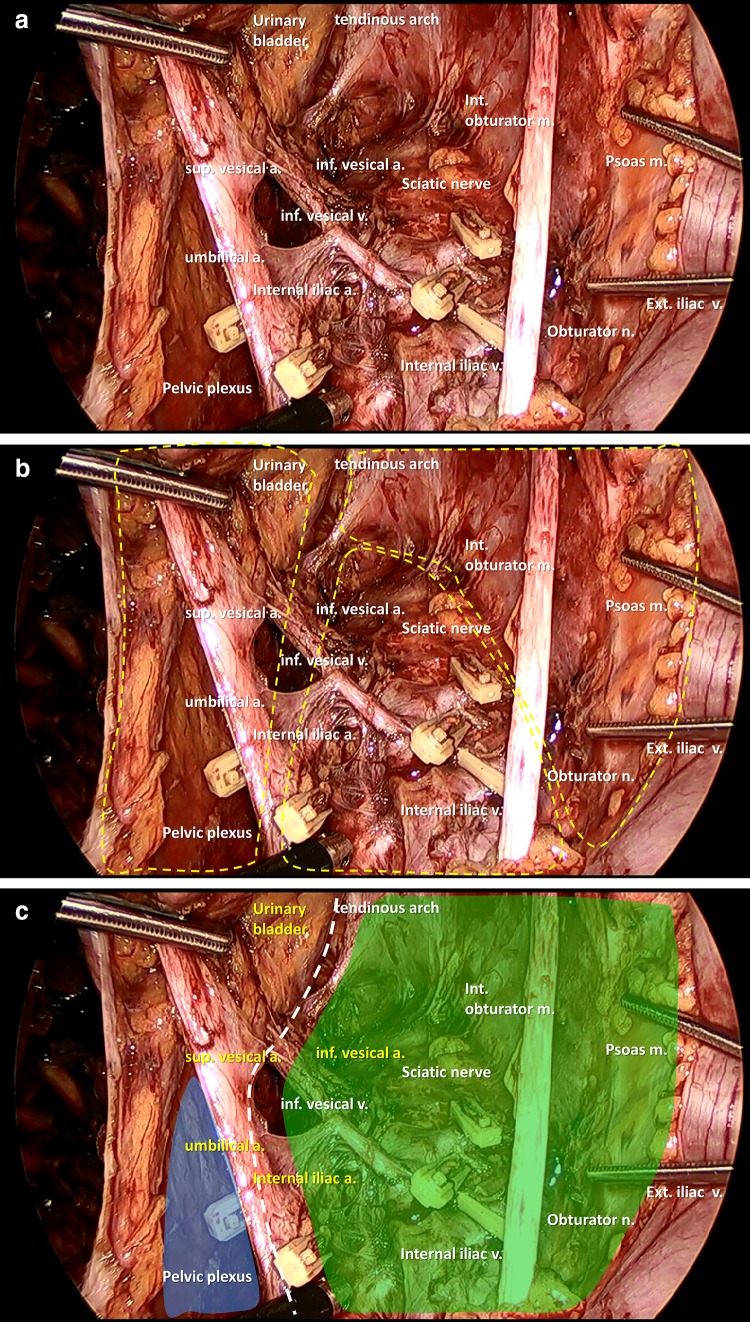
**a** Laparoscopic view of the anatomy of the lateral area after lateral node dissection. **b** Dissection planes for lateral node dissection. **c** Vesicohypogastric fascia (dotted line), which divides the lateral area into the obturator (blue) and internal iliac (green) compartments. Abbreviations: *sup* superior, *inf* inferior, *int* internal, *ext* external, *a* artery, *v* vein, *n* nerve, *m* muscle

The technical procedures of laparoscopic LPLND are well established and have been standardized by multiple authors [13, 36]. The important advantage of laparoscopic LPLND is a better surgical view within the deep pelvis, which allows for identification of the pelvic vessels and autonomic nerves (Fig. 2a). Typically, LPLND should be performed after completion of TME and before anastomosis. No additional trocars are needed after TME. The obturator and internal iliac nodes are the most important to dissect because they cover most of the curable lateral node metastasis from rectal cancer. To dissect these two areas, three planes should be recognized: the lateral pelvic wall plane, which is composed of the psoas and internal obturator muscles; the medial plane, composed of the ureter and the pelvic plexus; and the dorsal plane, composed of the internal iliac vessels and the sciatic nerve (Fig. 2b). These three planes surround the area to be dissected. Another important plane divides the area into the obturator and internal iliac compartments: the vesicohypogastric fascia, composed of the bladder, internal iliac artery, and the urinary branches (the umbilical and superior vesical and inferior vesical arteries; Fig. 2c). Dissection along these planes minimizes bleeding and prevents incomplete dissection in LPLND.

### Postoperative complications

The reported postoperative complication rates of laparoscopic and robotic LPLND range from 7 to 41% and 25 to 39%, respectively (Table 3) [13, 22–29, 32, 33, 35]. These rates are equal to or lower than the complication rates after open procedures [3, 26, 32]. Ogura et al. reported that the incidence of major complications (grade ≥ 3) after laparoscopic LPLND was 9.3%, including anastomotic leakage, pelvic abscess, ileus and postoperative bleeding [27]. Bae et al. reported a postoperative complication rate of 28% after laparoscopic or robotic LPLND, including anastomotic leakage, ileus and chyle leakage [25]. Kim et al. compared the short-term outcomes of laparoscopic and robotic LPLND and found that the incidences of postoperative complications were similar (28% vs. 34%, respectively; *P* = 0.63) [35]. Yamaguchi et al. reported that the rates of wound infection, small-bowel obstruction, and anastomotic leakage after robotic LPLND were lower than those after open LPLND (*P* < 0.05 for all) [32].

**Table 3:** Postoperative genitourinary dysfunction after lateral lymph node dissection for rectal cancer

Author	Year	Number of patients	ANP	Surgical procedure	Urinary function	Sexual function
Sugihara [38]	1996	214	Yes	Open		29.6% male sexual dysfuction (Bilateral ANP) 33.3% no erection (removal of the hypogastric nerves)
Matsuoka [39]	2001	83	N/A	Open	86% dysuria 40% urinary incontinence 25% need CIC for more than 3 years	
Maeda [40]	2003	65	Yes	Open	15% minor disturbance (25% without LPLND)	27% impotency (20% without LPLND) 11% retrograde ejaculation (25% without LPLND)
Col [41]	2005	24	N/A	Open	58% urinary incontinence (39% without LPLND) 16% urinary retention (4% without LPLND)	
Akasu [42]	2009	42	Yes/No	Open		44%, 44%, 100% no erection (Bilateral ANP, unilateral ANP, no ANP) 0%, 50%, 100% no ejaculation (Bilateral ANP, unilateral ANP, no ANP)
Saito [5]	2016	701	Yes	Open		79% sexual dysfuction (68% without LPLND)
Ito [6]	2018	701	Yes	Open	59% urinary incontinence (58% without LPLND)	
Liu [45]	2013	60	Yes	Laparoscopic	78% incomplete emptying 70% frequency	
Ogura [27]	2016	107	Yes	Laparoscopic	5% urinary retention requiring CIC (1.5% without LPLND)	
Yamaguchi [32]	2016	85	Yes	Robotic	18.8% and 36.4% urinary retention in robotic and open LPLND	
Kim [35]	2018	50	Yes	Robotic	4% and 20% urinary retention in robotic and Laparoscopic LPLND	

*ANP* autonomic nerve preservation, *N/A* not assessed, *CIC* clean intermittent catheterization, *LPLND* lateral pelvic lymph node dissection

Postoperative urinary and sexual dysfunction are major complications after rectal surgery and the addition of LPLND has been reported to result in more genitourinary dysfunction than TME alone [37–42]. However, nerve-preserving techniques minimize this dysfunction after LPLND [43, 44]. A recent Japanese RCT reported similar rates of sexual and urinary dysfunction after TME alone vs. TME plus LPLND through an open approach (male sexual dysfunction, 68% vs. 79%, respectively; *P* = 0.37; subclinical urinary dysfunction with ≥ 50 mL residual urine, 59% vs. 58%, respectively) [5, 6]. The authors concluded that if autonomic nerve-preserving procedures are done, LPLND does not increase the risk of sexual or urinary dysfunction. A few studies have investigated genitourinary dysfunction after laparoscopic and robotic LPLND. Liu et al. reported adequate urinary function after laparoscopic LPLND [45]. At a high-volume center in Japan with experienced laparoscopic surgeons, the incidence of postoperative urinary dysfunction was minimal [13, 27]. Manabe et al. reported that combined resection of the bilateral inferior vesical arteries was a risk factor for postoperative urinary dysfunction after laparoscopic LPLND [46]. It should be noted that not only the autonomic nerves but also the inferior vesical arteries are closely associated with functional outcomes after LPLND. Although the data are limited, robotic LPLND may allow for better handling of these structures, resulting in a lower incidence of postoperative genitourinary dysfunction than after open or laparoscopic LPLND [32, 35]. The risk of postoperative complications after LPLND is influenced by multiple factors, including whether the procedure is prophylactic or definitive and unilateral or bilateral. Case-matched prospective validation studies are needed to investigate this further.

## Conclusion

In the era of NACRT/RT, selective LPLND provides oncological benefits to patients with suspicious lateral nodes, particularly for reducing local recurrence. Careful patient selection and the appropriate use of minimally invasive surgery have the potential to improve short-term and long-term outcomes. Further studies are warranted to promote the minimally invasive approach for LPLND, including its technical feasibility in a larger dataset, complication profiles, leaning curve, continence, urinary/sexual function, and oncologic long-term outcomes.
